# Recommendations for Women in Mountain Sports and Hypoxia Training/Conditioning

**DOI:** 10.1007/s40279-023-01970-6

**Published:** 2023-12-12

**Authors:** Johannes Burtscher, Antoine Raberin, Franck Brocherie, Davide Malatesta, Giorgio Manferdelli, Tom Citherlet, Bastien Krumm, Nicolas Bourdillon, Juliana Antero, Letizia Rasica, Martin Burtscher, Grégoire P. Millet

**Affiliations:** 1 Institute of Sport Sciences, Faculty of Biology and Medicine, University of Lausanne, Building Synathlon, Campus Dorigny 1015 Lausanne, Switzerland; 2 Laboratory Sport, Expertise and Performance (EA 7370), French Institute of Sport, Paris, France; 3 Institut de Recherche Bio-Médicale Et d’Épidémiologie du Sport (EA 7329), French Institute of Sport, Paris, France; 4 Faculty of Kinesiology, University of Calgary, Calgary, AB Canada; 5 Department of Sport Science, University of Innsbruck, Innsbruck, Austria

## Abstract

The (patho-)physiological responses to hypoxia are highly heterogeneous between individuals. In this review, we focused on the roles of sex differences, which emerge as important factors in the regulation of the body’s reaction to hypoxia. Several aspects should be considered for future research on hypoxia-related sex differences, particularly altitude training and clinical applications of hypoxia, as these will affect the selection of the optimal dose regarding safety and efficiency. There are several implications, but there are no practical recommendations if/how women should behave differently from men to optimise the benefits or minimise the risks of these hypoxia-related practices. Here, we evaluate the scarce scientific evidence of distinct (patho)physiological responses and adaptations to high altitude/hypoxia, biomechanical/anatomical differences in uphill/downhill locomotion, which is highly relevant for exercising in mountainous environments, and potentially differential effects of altitude training in women. Based on these factors, we derive sex-specific recommendations for mountain sports and intermittent hypoxia conditioning: (1) Although higher vulnerabilities of women to acute mountain sickness have not been unambiguously shown, sex-dependent physiological reactions to hypoxia may contribute to an increased acute mountain sickness vulnerability in some women. Adequate acclimatisation, slow ascent speed and/or preventive medication (e.g. acetazolamide) are solutions. (2) Targeted training of the respiratory musculature could be a valuable preparation for altitude training in women. (3) Sex hormones influence hypoxia responses and hormonal-cycle and/or menstrual-cycle phases therefore may be factors in acclimatisation to altitude and efficiency of altitude training. As many of the recommendations or observations of the present work remain partly speculative, we join previous calls for further quality research on female athletes in sports to be extended to the field of altitude and hypoxia.

## Key Points

**Table:** 

The body’s responses to low oxygen availability (hypoxia) are characterised by increasingly recognised differences between women and men.
The consequences of these sex differences and female-specific responses are under-investigated; systematic future research on the safety and performance of women training or competing in hypoxia is required.
At present, a potentially higher vulnerability of female athletes to acute mountain sickness, specific preparation of the respiratory musculature, and the influence of sex hormones on hypoxia responses are important considerations for athletes and coaches.

## Introduction

Low oxygen availability (hypoxia) can massively affect human physiology [1, 2]. The hypoxic dose (i.e. combination of duration, severity, type and intermittent pattern of the exposure to hypoxia), environmental (e.g. temperature) and behavioural (e.g. exercise) conditions, as well as individual predispositions determine acute responses and long-term adaptations, which can be both protective or maladaptive/pathological [3, 4].

Although there is a great bias in the literature on hypoxia research towards male subjects, an expanding body of literature indicates potential sex-based differences in responses to hypoxia, including ventilatory [5, 6], cardiac [7], haemodynamic [8], muscle metabolism [9] and autonomic responses [10, 11]. Hormonal changes and the influence of the menstrual cycle and menopause may partially underlie those differences [12, 13]. The poor understanding of sex differences in hypoxia responses is contrasted by an increasing number of women engaging in leisure or competitive mountain sports (e.g. mountaineering, trail running, ski mountaineering, cross-country or alpine skiing, snowboarding), including at the Winter Olympics which are often performed at altitude (e.g. Cortina, Italy, 2026).

The public and scientific interest in hypoxia research is rapidly increasing. This is not only because of increasing travel to mountainous regions and growing numbers of people performing sports at high altitude [14], which are linked to more individuals exposing themselves to the risk of developing high-altitude illnesses (HAIs; general practical recommendations for minimising HAIs are available [15, 16]). There is also an increasing interest in altitude/hypoxic training in both endurance [17] and team-sports athletes [18]. In addition, the evidence on potential therapeutic benefits of controlled hypoxia exposure (e.g. intermittent hypoxia) is growing [4, 19]. Specifically, clinical applications for older people [20], hypertensive subjects [21], or persons with neurological [20] or psychiatric diseases [22] have been proposed. Little scientific information is, however, available on sex differences in hypoxia responses regarding HAIs, altitude training and hypoxia conditioning. Furthermore, there are no practical recommendations if/how women should behave differently from men to optimise the benefits or minimise the risks of these hypoxia-related practices. The need for more research on the specific responses of women to altitude, including in combination with other environmental stressors (cold, heat), was recently emphasised [23]. Here, we evaluate the scarce scientific evidence of distinct (patho)physiological responses and adaptations to high altitude/hypoxia, biomechanical/anatomical differences in uphill/downhill locomotion, which is highly relevant for exercising in mountainous environments, and potentially differential effects of altitude training in women. Based on these factors, we derive sex-specific recommendations for mountain sports and intermittent hypoxia conditioning, while indicating particularly important research questions regarding these topics.

The literature search was performed using PubMed, Google Scholar, Scopus and Web of Science with the combination of the following keywords: “woman/women”, “female”, “hypoxia”, “altitude”, “acclimatisation exercise”, “mountain sports”, “high altitude illnesses”, “acute mountain sickness”, “respiration” and “muscle”. Relevant publications in the English language were selected based on the following criteria: primarily human studies were considered, sex differences could be assessed either directly in the article or with comparable articles, articles could be used to extract practical recommendations for women engaging in exercise activities at high altitudes. The selected articles were complemented with publications from the repertoire of the individual authors according to their expertise. Evidence levels are estimations based on evaluation and discussion among the authors.

## Hormone Profiles of Women and Metabolic Implications

An important factor underlying physiological sex differences are sex hormones. Consideration of female sex hormone profiles therefore is also important when assessing the risks and opportunities of altitude training in women. Fluctuations of sex hormone levels (e.g. during the menstrual cycle or as a consequence of menopause) affect exercise metabolism [24] and modulate the physiological responses to hypoxia, in part due to their regulation of body temperature and respiratory and cardiovascular functions. From an age of about 13 years, eumenorrheic (“normal menstruation”, in contrast to amenorrhoeic) women experience the menstrual cycle, a relatively predictable rhythm of 23–38 days, characterised by typical fluctuations of the hormones follicle-stimulating hormone, oestrogen, progesterone and luteinising hormone [25]. Ovulation separates the two major phases of the menstrual cycle, the follicular phase and luteal phase, and follows a sharp increase in luteinising hormone, oestrogen and follicle-stimulating hormone levels during the follicular phase. During the luteal phase, oestrogen and progesterone levels progressively increase until the mid-luteal phase, when their levels start to slowly decline. These changes in hormones are associated with changes in energy metabolism, and substrate preferences, as summarised in detail elsewhere [24]. Briefly, high oestrogen levels during the luteal phase improve glucose metabolism by increasing glucose availability and glycogen storage in skeletal muscle and increasing the availability of free fatty acids and oxidative energy metabolism. The effects of oestrogen on glucose metabolism are partly antagonised by progesterone and a high dietary carbohydrate intake can super-compensate muscle glycogen stores during the early follicular phase. In addition, oestrogen has complex effects on the respiratory and cardiovascular system (see Sect. 3). Despite an increased ventilatory drive during the luteal phase, the menstrual phase appears to not significantly influence exercise performance at low altitudes [24]. However, women raised or permanently living at high altitudes have been shown to have a slightly increased aerobic exercise capacity during the luteal phase [26], an effect not shown in female lowlanders exposed to acute hypoxia [27].

Menopause starts on average at about 51 years of age and results in the absence of the menstrual cycle. This process is characterised by a gradual loss of oestrogen, which is partly compensated for by increasing levels of follicle-stimulating hormone. These changes can perturb glucose and fatty acid metabolism, affecting multiple organ systems, including adipose tissue, bones, the gut microbiome and skeletal muscles [28]. Although sex hormones due to their regulation of metabolism, respiration, and the cardiovascular system likely influence responses to hypoxia and potentially the development of HAIs, this topic is insufficiently understood.

## High-Altitude Illnesses

Acute mountain sickness (AMS) and high-altitude cerebral oedema (HACE) have been suggested to represent manifestations on a continuum of the cerebral form of HAIs [29–31]. The risk of AMS steeply rises when unacclimatised individuals are exposed to increasing altitude, affecting more than 50% of them at altitudes above 4500 m [31–35]. Fortunately, AMS only rarely (in about 1%) progresses to HACE [36]. While the course of AMS is usually benign and self-limited, HACE represents a life-threatening form of HAI associated with a 50% mortality when untreated [15, 35]. In this section, we review the existing literature on putative sex differences in HAIs.

The main physiological responses to acute high-altitude exposure include hyperventilation, haemoconcentration, sympathetic activation accompanied by a rise in heart rate and cardiac output, and in contrast to the peripheral and cerebral vasculature, pulmonary vasoconstriction and elevated pulmonary arterial pressure (PAP) [2, 4, 37–39]. If these responses are too slow and insufficiently compensate for high-altitude-induced hypoxemia, elevated intracranial pressure, brain swelling, and oedema formation may provoke the development of AMS or even HACE [40–44]. The activation and sensitisation of the trigemino-vascular system by both mechanical (e.g. intracranial pressure) and chemical factors (e.g. oxidative stress and inflammatory parameters) may cause headaches [31, 45–47], and in rare cases, HACE may result from dysfunction or disruption of the blood–brain barrier [43, 48, 49]. The extent and course of these (patho)physiological responses to acute high-altitude exposure differ between individuals and probably specifically between sexes [50, 51]. Such differences may contribute to disparities in AMS development between men and women. A recent meta-analysis demonstrated a higher AMS prevalence in women compared with men with 15 out of 18 studies in favour of this tendency (risk ratio = 1.24, 95% confidence interval = 1.09–1.41) [51]. This may be explained by oestrogen-mediated intracranial hypertension and/or a lower antidiuretic hormone threshold, increasing fluid retention, which is associated with severe AMS [51]. Importantly, whether women really are at higher risk for AMS is debated and several studies (including some in the aforementioned meta-analysis [51]) did not find such a difference [52, 53].

Mechanisms explaining the potentially higher AMS prevalence in women remain largely unexplored. In contrast to other studies [54], some evidence suggests that women may become more hypoxemic during the first hours at altitude/in hypoxia [50, 55]. Camacho-Cardenosa et al. report a less pronounced ventilatory response to acute moderate normobaric hypoxia (fraction of inspired oxygen F_I_O_2_ = 0.15) in women than men [50]. This was associated with a significantly lower peripheral oxygen saturation (SpO_2_) in women during the first 2.5 h of hypoxia exposure, followed by similar SpO_2_ in women and men thereafter [50]. A somewhat steeper SpO_2_ decline in women was also reported during submaximal exercise during the first hours at high altitude (3500 m) [55]. If this will be confirmed as a common phenomenon, more severe hypoxemia may provoke a larger increase in intracranial pressure in women, initiating AMS development. In addition, oestrogen may aggravate pathological responses by upregulating vascular endothelial growth factor expression [56], which could provoke vascular leakage, increase exudation of tissue fluid, and consequently intracranial hypertension and/or even HACE [57]. This hypothesis is supported by higher AMS and HACE rates demonstrated in female pilgrims [58].

Like HACE, high-altitude pulmonary oedema is a rare but life-threatening form of HAI, prompting rapid treatment (e.g. by oxygen, nifedipine or descent) [37, 59]. High-altitude pulmonary oedema is a non-cardiogenic pulmonary oedema occurring subsequent to hypoxic pulmonary vasoconstriction and the associated increase in PAP and pulmonary capillary pressure [59, 60]. Genetic predisposition may favour a pronounced pulmonary vascular response (vasoconstriction) to hypoxia that seems to be accompanied by insufficient bioavailability of nitric oxide, likely related to exaggerated production of reactive oxygen species [61, 62].

Importantly, there is an inverse relationship between SpO_2_ (as well as arterial oxygen saturation, S_a_O_2_) and PAP [63, 64]. An initially (but likely transient) more pronounced reduction of SpO_2_ following hypoxia exposure in women (as suggested above) may result in elevated pulmonary vascular resistance and PAP. This assumption is supported by the findings of Fatemian et al. [65], but not by another study, in which individuals of both sexes were exposed to acute normobaric hypoxia equivalent to 4800 m [7], likely indicating different responses evoked by hypobaric and normobaric hypoxia [66, 67]. A larger high-altitude pulmonary oedema prevalence was observed in female pilgrims [58], which may be considered supportive of more severe hypoxia and higher PAP occurring in women during acute high-altitude/hypoxia exposure. A potential role of oestrogen for provoking or preventing high-altitude pulmonary oedema development and related consequences remains to be elucidated. Pulmonary hypertension occurs more frequently in women than men even though oestrogen was demonstrated to elicit beneficial effects on the pulmonary vasculature [68]. This “oestrogen paradox” may be explained by the complex and different effects exerted by endogenous and exogenous oestrogen and peripheral oestrogen metabolites [69]. However, in contrast to the varying effects of oestrogen on the pulmonary vasculature, oestrogen has been consistently found to promote favourable functioning of the right ventricle [70].

Finally, several studies demonstrated an increased risk of men dying from sudden cardiac events during exercise at low and high altitudes as well [71, 72]. Mechanisms explaining these sex differences are still not well understood. In addition to sex-specific differences in exercise behaviours [72], it seems likely that sex hormones (i.e. oestrogen) may contribute to some protection against illnesses, such as atherosclerosis and related coronary events [71, 73].

## Uphill/Downhill Locomotion

Altitude exposure is often synonymous with a mountainous environment that requires locomotion on positive (uphill) or negative (downhill) slopes. While graded locomotion is extensively investigated [74–80], the literature investigating whether there are sex differences in the energetics and biomechanics of incline walking and running is scarce [81–86]. Women showed a higher stride frequency, a shorter stride length, greater non-sagittal hip and pelvis motion (i.e. higher peak hip internal rotation and adduction) and similar sagittal motion compared with men [81, 82]. Moreover, women displayed greater gluteus maximus activity during level and incline walking (1.2, 1.5 and 1.8 m/s at 0, 10 and 15%) and running (1.8, 2.7 and 3.6 m/s at 0, 10, and 15%) [82]. Gluteus medius and vastus lateralis activities increased more with speed and incline, respectively, in women than in men [82]. Overall, these results corroborate that men and women use different neuromuscular strategies with increasing efforts during walking and running at faster speeds or steeper inclines [82]. It has been shown that these biomechanical sex differences disappear when corrected for height or body mass or tested at the same relative walking and running speeds (in percent of maximal oxygen uptake [*V*O_2max_] or maximal aerobic speed) [81], suggesting that the observed biomechanical differences are mainly due to sex-related morphology differences (e.g. women have a lower body size on average).

Regardless of these sex differences in walking and running biomechanics, the energy cost of level and incline walking and running per kilogram of body mass transported (i.e. the energy expenditure per unit of distance; the walking/running economy) has been frequently reported to be similar between men and women [81, 83–85]. Therefore, some authors suggested that both sexes are able to optimise their walking/running patterns according to their own characteristics [81]. However, the sex differences in walking and running economy remain controversial. In walking, there were no differences between sexes in the mass-normalised energy cost at slopes of 0% and 5%, whereas this parameter was higher in women than in men during walking at slopes of 10% and 15% [83]. This difference may be due to (1) the smaller size, (2) higher body mass distributed peripherally and (3) greater upper limb movements during walking in women compared with men [83]. In level running, Mendonca et al. [87] recently reported that the energy cost allometrically scaled by body mass assessed during running at absolute (8–12 km/hour) and relative (80–95% of *V*O_2max_) speeds was lower in women than in men matched for age and level of aerobic fitness (i.e. similar percent difference from predicted *V*O_2max_). Importantly, these results were obtained by testing women in the early follicular phase of their menstrual cycle, controlling the likely effect of the latter on running economy. These authors suggested that, from a performance perspective, the higher running economy may partially compensate for the lower *V*O_2max_ in female runners [87]. However, controversial findings exist on sex differences in fatigue resistance and pacing in road and trail ultra-marathons [81, 88, 89]. Some authors reported greater fatigue resistance and better pacing in female ultra-marathon participants [81, 89], whereas others found that women tended to slow down more than men in the later stages of trail running ultra-marathons, despite the terrain (uphill and downhill) [88]. Further studies are needed to better understand sex differences in the energetics and biomechanics of incline walking and running, and fatigue resistance and pacing strategies in ultra-marathons controlling for fitness levels, anthropometric differences, menstrual cycle effect and the level of relative effort used for testing both sexes.

Overall, acute altitude exposure (i.e. altitude training camps) seems not to alter the energetics (i.e. energy expenditure above resting per distance unit: running economy) and biomechanics (i.e. spatiotemporal, kinematic and kinetic parameters used to assess gait pattern) of running in male runners [90]. Although chronic altitude exposure in native altitude male runners has been suggested to contribute to the higher running economy in East African runners than in European runners [91, 92], this is controversial [93], with a lack of evidence investigating the specific biomechanical determinants and sex differences.

## Altitude/Hypoxic Training

Athletes commonly use altitude/hypoxia as a stimulus to induce physiological adaptations in order to prepare for competitions at altitude or to improve sea-level performance [94–97]. Historically, altitude/hypoxic training was popularised among endurance athletes and typically involved chronic hypobaric or normobaric hypoxic exposure such as “Live High-Train High” or “Live High-Train Low” (LHTL) strategies [98]. More recently, the development of “Live Low-Train High” or intermittent hypoxia interventions [99] has affected the evolution of the panorama of altitude/hypoxic training [100]. However, studies specifically reporting effects on women are scarce and, with few exceptions, mostly focused on men or lacked specific comparisons between sexes. In this section, we describe the putative differences in the effect of altitude/hypoxic training between men and women.

In acute hypoxia, aerobic performance is altered in both trained and untrained men, with a larger drop in *V*O_2max_ in male athletes than in sedentary individuals [101–104]. Women (and particularly highly trained women) may experience more severe respiratory limitations during exercise than men [5, 6, 105–107] and they appear to be more susceptible to exacerbated exercise-induced arterial desaturation and hypoxemia (67% of healthy young women [108] vs 0% of untrained or moderately trained men and 52% of male elite athletes [109] were reported to be hypoxemic during maximal cycling exercise). This is thought to be due to higher work of breathing and reduced diffusion capacity in women during maximal exercise in acute hypoxia compared with men [5, 6], resulting in mechanical ventilatory constraint and/or an inadequate ventilatory drive. While some studies did not detect sex differences in the *V*O_2max_ decline with a gain in altitude [110], sufficient hyperventilation may actually be more limited in women than men because of a more pronounced ventilatory restriction [111]. This effect was indeed observed in women [112, 113], with a significantly larger decrease in *V*O_2max_ in trained women than untrained women above 2500 m [113, 114]. Consideration of hormonal status and training status/type of exercise for the evaluation of sex differences in altitude training will be crucial in future studies. In line with a previous observation showing that elite female endurance athletes already exhibit altered *V*O_2max_ at an altitude of 580 m [101], a greater decrease in VO_2max_ in endurance-trained women versus untrained women is due to a lower S_p_O_2_ at maximal exercise [113] resulting from diffusion limitation [114, 115]. Interestingly, the decrease in *V*O_2max_ was smaller in women than in men during 6–7 days trekking at 4350 m [116], suggesting that hormones represent one factor determining the sensitivity to hypoxia [13, 117].

The primary aim of altitude/hypoxic training at moderate altitudes (e.g. “Live High-Train High” or LTHL at 2000–2500 m) is to stimulate erythropoiesis and subsequent haematological adaptation [118–124], leading to enhanced *V*O_2max_ and competitive performance [125]. Although the hypoxia-induced individual erythropoietic response is highly variable [123, 126], for every 100 h of either hypobaric or normobaric hypoxic exposure over a minimum of 2 weeks [120, 127], athletes may achieve a ~ 1.0–1.1% increase in total haemoglobin mass (Hb_mass_). This dose–response relationship may be sex dependent [128–130]. For instance, a significant increase was observed in both erythropoietin [EPO] (31%) and reticulocyte count (5%) after 11 days of LHTL at 2500 m in six female elite cross-country skiers [131]. Similarly small but relevant LHTL dose–response improvements were reported in Olympic level female water polo athletes [132]. In contrast, six female road cyclists who underwent 12 days of LHTL (night at 2650 m and training at 600 m) exhibited no significant changes in haematological variables (i.e. reticulocyte count, mean corpuscular haemoglobin, reticulocyte haemoglobin and Hb_mass_) [133], with questionable effects on performance (i.e. + 2.3% and − 1.1% changes in 4-min and 30-min time-trial mean power output after LHTL vs + 0.1% and + 2.4% in sea-level controls) [134]. Of note, the Hb_mass_ increase at moderate altitude (2600 m) was smaller in women (+ 6.6%) than in men (+ 12%) at equal peak oxygen uptake, although different group sizes and categories (i.e. trained vs untrained) in the separately performed studies on men and women complicate interpretation [128–130].

Numerous confounding factors such as altitude type (i.e. hypobaric vs normobaric), hypoxic dose (i.e. duration and level of exposure) [120, 135–138] and possibly initial Hb_mass_ level [139], iron deficiency, illness, inflammation or insufficient energy availability [140] are supposed to blunt the erythropoietic response to altitude exposure and consecutive haematological adaptation [141–144]. The cyclic variation in sex hormones [145, 146] also plays a role in the regulation of EPO production in hypoxia [147]. During the menstrual cycle, the large change (approximately ten-fold) in estradiol [148], an EPO inhibitor [149], and the significant but probably not clinically relevant increase in testosterone [150], which is well known to promote EPO production [151], likely alter the hypoxia-induced erythropoietic response [147]. Possible protective effects of oestrogen and progesterone from oxidative damage [152] may also play a role in the sex difference in hypoxia tolerance and subsequent haematological adaptations [117, 129]. Although several studies did not report differences in Hb_mass_ response to altitude between sexes [153, 154], this could be because of statistical flaws (e.g. a comparison involving smaller numbers of women) [122, 142, 154–156]. In contrast, Heikura et al. [157] recently reported higher relative and percentage Hb_mass_ increases in women, compared with men. They further found lower pre-hypoxic exposure Hb_mass_ levels in amenorrheic versus eumenorrheic women, suggesting that menstrual dysfunction, an indicator of long-term low energy availability, may influence these adaptations or their magnitude [157]. Of note, whereas menstrual blood loss has no measurable effect on Hb_mass_ across phases of the menstrual cycle in eumenorrheic women, oral contraceptive use, which increases serum iron levels by decreasing menstrual blood loss, contributes to greater oxygen-carrying capacity and possibly greater *V*O_2max_ [145]. Overall, the consequences and safety of different types of hormonal contraceptives (progestin only and combined) in relation to athletic performance, particularly in combination with hypoxia, require further scrutiny. This is becoming more important with the increasing use of hormonal contraceptives by female athletes to prevent perceived menstrual-linked impairments of training or competition performance [158]. A hypoxia-induced Hb_mass_ response may not be detectable because of an insufficient hypoxic dose [137] or limited potential for adaptation [159]. Relevant Hb_mass_ and performance enhancement [160] may still remain possible, even if baseline Hb_mass_ levels are already high [139]. Moreover, other non-haematological adaptations to hypoxia (e.g*.* running economy, glycolysis and buffering capacity) may occur independently of Hb_mass_ change [160, 161]. Given the wide intra-individual and inter-individual variations at altitude/in hypoxia response [123] and the uncertain evidence regarding peak performance timing (likely dependent on the combination of acclimatisation to altitude training camps and subsequent deacclimatisation responses) [118], specific periodisation and individualisation of training are critical aspects to consider [162]. These factors are likely affected by physiological sex-based differences.

Another means to improve performance is “Live Low-Train High”. In “Live Low-Train High”, the low hypoxic dose is unlikely to enhance Hb_mass_ but the combination of hypoxic stress with high-intensity interval exercise plays a role on adaptations at the molecular level in skeletal muscle tissue (e.g. mitochondrial efficiency and pH/lactate regulation) [98, 163–167]. Aerobic training in chronic hypoxia in female trekkers did not induce substantial mitochondrial benefits (mitochondrial biogenesis, mitochondrial respiration) or improvements in muscle fibre composition (including distribution of muscle fibre types I and IIA/IIX) [168]. However, hypoxia exposure in young eumenorrheic women induced an, at least partially, α-adrenergic pathway-mediated, exercise-independent upregulation of interleukin-6 (a stress response well known for exercise) [169], with haematological changes related to the immune system [170, 171]. Similarly, a blood-related signature of hypoxic high-intensity exercise was reported in elite female speed skaters [172]. In this study, the major changes associated with hypoxic exercise were related to innate immune responses (inflammation), the hypoxic stress response and platelet activity. In amateur Korean women runners, 6 weeks of intermittent hypoxia training (3000 m) improved endurance performance; concomitantly, the oxygen-carrying capacity (although not related to erythropoiesis) and haemodynamic functions were improved, with immune system-related haematological parameters remaining in the “normal” range [173]. Similarly, repeated-sprint training in hypoxia (RSH) did not impair mucosal immune function [170], while providing putative performance benefits [174]. In the absence of direct comparisons of RSH effects between women and men, and based on the lower sensitivity of women to hypoxia compared with men [175], the effect of RSH might be smaller in female athletes. To date, only a few studies [176, 177] have investigated RSH in female athletes. Four weeks of RSH (2 × 10 × 7-s sprints with 30-s rest periods between sprints; F_I_O_2_ = 0.145; twice per week) did not modify *V*O_2max_ but resulted in an about a three-fold greater increase in peak power output, as well as power output during all sprints, compared with similar training in normoxia [177]. It was hypothesised that power output impairment during RSH would be greater among women than men because of their higher proportion of type-1 oxidative fibres and anaerobic contribution [178]. However, a marked power output decrease was noticeable [176] and accompanied by large increases in blood lactate concentrations during RSH, suggesting that glycolytic metabolism was augmented under hypoxia in women [176]. A direct sex comparison was recently performed by Paez et al. [6], who reported a lower tolerance to anaerobic glycolysis observed in women versus men performing repeated sprints (30 s full effort and 20 s recovery until failure) either in hypobaric hypoxia (3264 m) or in normoxia [6]. A negative energy balance and unfavourable iron status (baseline s-ferritin < 20 μg·L^−1^ for women and < 30 μg·L-1 for men) may decrease exercise performance, physical, and health conditions particularly in women and may perturb the menstrual cycle [179]. Accordingly, further research is warranted to clarify sex differences in performance and physiological variables (e.g., SpO_2_, metabolites and endocrine responses) during RSH. This is important for potential future applications (e.g. managing weight and preventing obesity in women) [180, 181].

Finally, resistance training in hypoxia can lead to structural and functional skeletal muscle adaptations, but potential sex-based differences have not been investigated [182]. While no difference was found between women and men performing squat and bench press at both maximal (i.e. one-repetition maximal) and submaximal (i.e. 60% one-repetition maximal) intensity in hypoxia (2000 m and 3000 m) compared to normoxia [182], whether the higher fatigue resistance reported in women [183, 184] would affect training remains to be clarified. For all altitude/hypoxic training methods, more studies are needed to describe and quantify the sex-based physiological effects (e.g. morphological, biochemical) and their possible dose–response relationships.

## Intermittent Hypoxia: Hyperoxia Conditioning

The interest in the application of protocols consisting of intermittent periods of mild hypoxia for preventive or therapeutic purposes has increased substantially during the past few decades [185]. Such protocols typically comprise cycles of repeated (usually three to six times per cycle), short (several minutes) hypoxia exposures, usually with F_I_O_2_ between 0.10 and 0.13. The cycles are often applied about 15–20 times across several (3–6) weeks, with a maximum 1 session per day. The short periods of hypoxia are interspersed with either normoxic phases of similar duration or hyperoxic phases, the latter possibly improving recovery from hypoxic stress and through additional benefits via the induction of complementary adaptations [19, 186].

The efficiency demonstrated for specific intermittent hypoxia protocols to counteract a cognitive decline in ageing [20], neurological and psychiatric diseases [20, 22], regeneration of the nervous system after brain and spinal cord injury [187], improved cardiovascular and ventilatory functions [2, 188] and ameliorated sleep-disordered breathing [187] is based on numerous cellular and systemic responses and adaptations to hypoxic stress. Among the probably most important mechanisms contributing to benefits of therapeutic/preventive intermittent hypoxia are cardiovascular adaptations (leading, for example, to reduced blood pressure in male patients with hypertensive obstructive sleep apnoea [189]), respiratory and autonomic plasticity [187], metabolic adaptations and regulation of inflammation at the systemic level [4]. At the cellular level, reduced reliance on oxygen in energy metabolism, reduced oxidative stress and increased resilience to hypoxic insults are major beneficial effects [4, 190].

Whether intermittent hypoxia elicits those beneficial effects or results in injury depends largely on the hypoxic dose and individual vulnerabilities [4, 190]. Severe intermittent hypoxia associated with diseases such as obstructive sleep apnoea does not lead to beneficial adaptations but to maladaptation and cellular damage. Obstructive sleep apnoea prevalence is higher in men compared with women [191–193]. In women, the prevalence increases with age, especially after menopause [191, 193]. Hormonal replacement therapy is associated with reduced obstructive sleep apnoea prevalence in post-menopausal women, indicating that female sex hormones play a protective role [191].

Potential differences in the responses to intermittent hypoxia interventions in terms of efficiency and safety between men and women are insufficiently explored, as most studies were either performed in only male or female individuals or no comparisons between male and female study participants were conducted. Such differences are, however, suggested by several recent findings that require confirmation for the individual selection of optimally calibrated protocols.

Recently, a more severe hypoxemia in response to 5 min of hypoxia (F_I_O_2_ = 0.10) in older women [194] and after 7 h of F_I_O_2_ = 0.15 in young women [50] compared with age-matched men has been reported. This observation is in agreement with previously described differences in oxygen transport [195] and in the respiratory system, including smaller conducting airways relative to lung size [196], which may impair ventilation in exercise conditions faster in women than in men. Consequentially, this may cause more severe hypoxemia during exercise in women [197]. The more pronounced changes in ventilation may be related to a more severe effect of the hypoxic conditions at high altitudes on nocturnal periodic breathing in men compared with women [198]. However, other studies did not observe major sex differences in ventilation in hypoxia. Wadhwa and colleagues observed similar increases in minute ventilation in men and women after eight 4-min episodes of hypoxia (end tidal partial pressure of oxygen maintained at 50 mmHg) interspersed with 5 min of normoxia (end tidal partial pressure of oxygen = 100 mmHg) [199]. Conversely, these authors observed sustained depression of parasympathetic nervous system activity and increased sympathovagal balance in men after hypoxia, which was not evident in women [199]. A comparison of physiological responses to high altitude (3480 m) after an exposure time of 2–5 h further revealed significant increases in blood pressure both at rest and during exercise in men (aged 22–67 years) but not in women (aged 20–61 years) [200]. Consideration of these physiological differences is important for study designs for intermittent hypoxia applications.

In conclusion, intermittent hypoxia protocols for preventive or therapeutic purposes hold great promise but sex differences in efficiency and safety are expected based on different responses to hypoxia in men and women, which has not been systematically assessed yet. In addition to differences in physiological responses, sex differences in prevalence, pathology, medication and symptoms also have to be taken into account when selecting optimal intermittent hypoxia protocols for therapeutic purposes in specific patient populations, as recently discussed for ischaemic stroke [201].

## Practical Considerations for Mountain Sports

Despite the above-described distinct (patho)physiological responses of women to hypoxia, a translation into practical considerations and recommendations to support female athletes and mountaineers to better acclimatise, prepare and perform in mountain sports is only possible to a limited degree. This is because of the scarcity of well-controlled studies specifically comparing differences in hypoxia responses in women and men.

Several high-quality reviews have been published on the potential sex differences in ultra-marathons [81, 89, 202]. As they did not focus on mountain ultra-marathons, potential influences of the sex differences in response to altitude/hypoxia were not discussed in those reviews. In a recent study on sex differences in mountain ultra-marathon athletes [203], the ventilatory and pulmonary limitations due to “intermittent altitude” were discussed. Recently, an interesting review on the potential sex differences to altitude combined with other stressors (heat, cold) has been published [23] and the authors argued that how this translates to performance or health outcomes remains under-investigated. To our knowledge, there are no comprehensive practical recommendations based on the physiological sex differences in responses to hypoxia.

Therefore, in this section, we aim to derive practical recommendations relating to specific responses to altitude/hypoxia in women (e.g. respiratory limitation, iron deficiency, prevalence of AMS, lower hypoxia-induced vasoconstriction, greater hypoxemia and a decrease in *V*O_2max_, higher hypoxic ventilatory response during the luteal phase, different shift in substrate preference, different muscle composition) which, however, require further scientific substantiation. Table 1 summarises these applications and three estimated levels of evidence (i.e. high, moderate and speculative, which require further exploration) are indicated.

**Table 1:** Specific characteristics of women in hypoxia and recommendations in mountain sports

Differences in women vs men	Consequences/recommendations for women	References	Evidence
Ventilation, larger expiratory limitations	Respiratory muscle training prior to or during altitude/hypoxic sojourn may be more beneficial	[106]	***
Higher risk of iron deficiency	Early and appropriate iron supplementation prior to and during altitude/hypoxic sojourn	[207]	***
Potentially higher risk for AMS	Pre-acclimatisation, slow ascent and potentially pharmacological prevention (acetazolamide). A chemosensitivity test could be considered	[51]	**
Lower vasoconstriction Greater vasodilation	Lower risk for sleep apneas or hypertension in prolonged exposure to altitude/hypoxia Possibly increased health benefits from hypoxia conditioning	[10]	**
Greater hypoxemia and decrease in *V*O_2max_	Monitoring of hypoxemia and appropriate reduction of training intensity during chronic altitude/hypoxia exposure	[194]	**
HVR higher during luteal phase	Possible reduced risk for AMS during the luteal phase?	[12]	*
Lower increase in CHO reliance	Lower risk of hypoglycaemia? Lower requirement for increased carbohydrate intake at altitude?	[221]	*
Muscle composition, more slow-twitch fibres	Effects on improvement in economy and on RSH	[222]	*

*AMS* acute mountain sickness, *CHO* carbohydrate, *HVR* hypoxic ventilatory response, *RSH* repeated-sprint training in hypoxia, *VO*_*2max*_ maximal oxygen uptake.

Level of evidence: *** high; ** moderate; * speculative

### Respiratory Muscle Training During Pre-acclimatisation

Before travelling to a high altitude, several pre-acclimatisation strategies using intermittent hypoxia exposures have proven effective for eliciting ventilatory acclimatisation (e.g. a decrease in end-tidal PCO_2_ due to hypoxia-induced hyperventilation and an increase in SpO_2_). Such a pre-acclimatisation strategy may be more effective when performed in hypobaric than in normobaric hypoxia [204], with the ventilatory benefits lasting for several days [205, 206]. However, the differences in physiological responses to hypobaric versus normobaric hypoxia remain insufficiently understood and depend strongly on the hypoxic dose. Because women elicit a larger expiratory flow limitation during hyperventilation [106], this suggests that combining muscle respiratory training and hypoxic pre-acclimatisation methods could be more beneficial in women than in men. One may speculate that the ventilatory benefits could improve performance specifically for maximal-intensity exercise at a high altitude.

### Higher Risk of Iron Deficiency

Adequate pre-altitude iron stores are needed for haematological adaptations during altitude exposure [96, 119] and women are at higher risks of iron deficiency [207]. Therefore, monitoring iron profiles prior to altitude exposure can be useful and is an important factor in training/performance optimisation in elite athletes [208]. Early checks (i.e. 6 weeks prior to an altitude sojourn) are recommended for female athletes. Systematic large iron supplementation (210 mg daily) in female endurance athletes has been suggested [119], although the optimal level of iron supplementation in athletes with clinically normal iron stores remains a subject of debate [209].

### Higher Prevalence of AMS

As women appear to have a statistically higher AMS risk (see Sect. 3), systematic screening of AMS based on the Lake Louise Scoring system is recommended. The Lake Louise Scoring system is a self-assessment questionnaire, rating the severity (no discomfort = 0; mild symptoms = 1; moderate symptoms = 2; severe symptoms = 3) of the following criteria: headache, nausea, dizziness and fatigue [210]. For women without any previous mountain experience, a chemosensitivity test (i.e. the assessment of the relationship between pulmonary ventilation and SpO_2_) prior to the ascent can also be an indication about their physiological responses to hypoxia [36, 211]. For those who have access to altitude/hypoxic facilities, long pre-acclimatisation (about 2 weeks with > 8 h/day at progressively increasing altitudes) is preferable to shorter exposures [204]. A slow ascent (< 400 m/day) further reduces the AMS risk.

### Lower Vasoconstriction and Larger Vasodilation

Sympathetic activation, a pivotal response to altitude [212, 213], has important pathophysiological consequences and regulates vasoconstriction/dilatation. Women frequently exhibit lower vasoconstriction and greater hypoxia-induced vasodilation [8]. This could mean that they are at a lower risk of increasing blood pressure or sleep apnoea during prolonged exposure to altitude/hypoxia. Conversely, the health-related vascular benefits of therapeutic hypoxia treatment [4] may be larger in women than in men for a similar hypoxic dose.

### Greater Hypoxemia and Reduction in ***V***O_2max_

The potential sex difference regarding the risk for hypoxemia of women during exercise in hypoxia requires consideration for high-altitude training but needs to be confirmed by future research. In the case of pronounced hypoxemia, training intensity may have to be decreased more in women than in men during chronic altitude/hypoxic exposure. However, as no direct comparisons of this effect in women and men under consideration of training status (an important determinant of exercise-induced hypoxemia, see Sect. 4) are available, the possibly sex-dependent association of altitude/hypoxic training camp/sojourn and hypoxemia requires further investigation. Based on the current state of knowledge, the recommendations of strict control of exercise intensities, particularly during the first days (i.e. acclimatisation phase), appear to be particularly relevant for women [98, 214].

### Higher Hypoxic Ventilatory Response During the Luteal Phase

There are several reports suggesting a higher hypoxic ventilatory response during the luteal phase [27, 215] favouring better oxygenation at a high altitude [13]. In theory, this would support the assumption that the mid-luteal phase would be the most appropriate timing for an acute exposure to a high altitude (i.e. summiting a high peak), while hypoxic tolerance tests should be performed in the follicular phase (when hypoxic ventilatory response is the lowest) to minimise the risk during subsequent exposure. Further work is required to confirm the relevance of these observations.

### Lower Increase in Carbohydrate Reliance at Altitude/in Hypoxia

Altitude exposure changes substrate oxidation for a given exercise intensity. It reduces the reliance on lipids and increases dependence on carbohydrate oxidation during exercise [216, 217], with a left shift of the cross-over point, i.e. power output at which energy from carbohydrate-derived fuels predominates over energy from lipids [218]. These shifts with greater carbohydrate utilisation at high altitudes [219] have nutritional consequences, such as greater dietary carbohydrate requirements to replace muscle glycogen and prevent hypoglycaemia during exercise [208, 220]. Women are less sensitive to this substrate shift [221] and therefore may require a smaller increase in carbohydrate intake at altitude. However, this may depend on the sex-hormonal status, for example, owing to the important regulation by oestrogen of glucose and fatty acid metabolism (see Sect. 2).

### Different Muscle Composition with Higher Slow-Twitch Fibre Proportion

There are probably several consequences of sex differences in muscle composition [222] for altitude/hypoxia training. Although speculative at this stage, a potentially improved economy (i.e. lower oxygen consumption at a given velocity) in response to altitude-induced changes in energy metabolism may differ between women and men. Such an improvement in economy has been reported following altitude exposure [223, 224] but, to our knowledge, there is no direct comparison between sexes.

Another putative consequence may be to determine the optimal type of RSH sessions (see Sect. 5). It is known that hypoxia reduces the power output during RSH at a higher oxidative but not glycolytic contribution [225]. With more slow-twitch fibres, the oxidative-glycolytic balance that is influenced by sprint duration and the exercise:rest ratio may be different between women and men. One may hypothesise that, in female athletes, RSH would require a less severe hypoxic stimulus to induce the expected peripheral benefits [226].

### Summary

Unfortunately, the scientific literature on altitude/hypoxia training does currently provide only limited information on sex differences affecting exercise in hypoxia. The eight points proposed above thus remain largely speculative and are important avenues for further research.

Future studies should consider the fitness level, matching women and men for training status and maximal oxygen consumption with adequate normalisation per kilogram of body mass or fat-free mass. It is recommended to follow adequate study designs to investigate sex differences in responses to altitude/hypoxia and exercise performance [227, 228].

## Conclusions

In this review, we overviewed the potential implications of sex differences in responses to altitude/hypoxia on performance and health. To our knowledge, this is the first attempt to translate scientific findings into practical recommendations.

The (patho-)physiological responses to altitude/hypoxia are highly heterogeneous between individuals. They determine the development of HAIs and the outcome of altitude/hypoxic training or hypoxia conditioning on performance and health and thus constitute important topics for research and applications in sports and clinics. Despite that, what constitutes inter-individual differences is poorly understood. Clearly, certain pathologies (especially respiratory diseases, such as chronic pulmonary obstructive disease), the individual genetic make-up, age and fitness modulate responses to hypoxia, particularly if combined with exercise. Here, we focused on the role of sex differences that emerge as important factors in the regulation of the body’s reaction to hypoxia. Some of the most relevant of these differences are summarised in Table 1. These factors should be considered for future research on hypoxia-related sex differences, particularly if altitude training and clinical applications of hypoxia are concerned, as they will affect the selection of the optimal hypoxic dose regarding safety and efficiency. Despite the poverty of scientific evidence on the topic, there are still several implications from which recommendations can be derived:

1. Various sex-dependent (patho)physiological reactions to hypoxia could explain the potentially increased vulnerability of women to develop AMS. Adequate acclimatisation, slow ascent speed and/or preventive medication, (e.g. by acetazolamide) are solutions.
2. Targeted training of the respiratory musculature could be a valuable preparation for altitude training in women.
3. Sex hormones influence hypoxia responses and hormonal-cycle and/or menstrual-cycle phases and therefore may be factors in acclimatisation to altitude and efficiency of altitude/hypoxic training. This should be considered for altitude sojourns and/or training but especially for future research.

As many of the recommendations or observations of the present work remain partly speculative, further quality research on female athletes is required not only for sports in general [146, 227, 228] but also for sports at altitude/in hypoxia.
